# Tertiary hospitals physician’s knowledge and perceptions towards antibiotic use and antibiotic resistance in Cameroon

**DOI:** 10.1186/s12879-021-06792-3

**Published:** 2021-10-29

**Authors:** Sandra C. Domche Ngongang, Wisdom Basera, Marc Mendelson

**Affiliations:** 1 Division of Infectious Diseases and HIV Medicine, Department of Medicine, University of Cape Town, Cape Town, South Africa; 2 School of Global Public Health, New York University, New York, USA; 3 School of Public Health and Family Medicine, University of Cape Town, Cape Town, South Africa; 4 Burden of Disease Research Unit, South African Medical Research Council, Cape Town, South Africa

**Keywords:** Antibiotic resistance, Antibiotic use, Knowledge and perceptions, Medical doctors

## Abstract

**Background:**

Infections due to resistant bacteria are associated with severe illness, increased risk for complications, hospital admissions, and higher mortality. Inappropriate use of antibiotics, which contributes to increased antibiotic resistance (ABR), is common in healthcare settings across the globe. In Cameroon, antibiotics have been reported as high as 45–70% of prescriptions. We sought to investigate the knowledge, attitudes, and perceptions regarding appropriate antibiotic use and ABR of medical doctors practicing in tertiary hospitals in Yaoundé, Cameroon.

**Methods:**

We conducted a cross-sectional survey using a 54-item self-administered questionnaire sent via email to medical doctors working in the four major tertiary hospitals of Yaoundé. The questionnaire recorded socio-demographics, perceptions on antibiotic use and ABR, sources and usefulness of education on ABR, and clinical scenarios to appraise knowledge.

**Results:**

A total of 98/206 (48%) doctors responded. Years of experience ranged between 1 and 17 years. Most participants agreed that ABR is a problem nationwide (93%) and antibiotics are overused (96%), but only one third (32%) thought that ABR was a problem in their wards. Most respondents (65%) were confident that they use antibiotics appropriately. We found a mean knowledge score of 56% (± 14), with prescribers not influenced by patient-exerted pressure for antibiotic prescribing scoring better compared to those influenced by patients (67% vs 53%, p = 0.01). Overall, most participants (99%) expressed interest for further education on both appropriate antibiotic use and ABR.

**Conclusion:**

Confidence of prescribers in their ability to appropriately use antibiotics conflicts with the low level of knowledge on antibiotic use in this group of doctors. Moreover, the opinion of the majority, that ABR is not a problem in their own backyard is in keeping with similar studies in other countries and is of significant concern. Introduction of formal antibiotic stewardship programmes in Cameroon may be a useful intervention.

## Background

The threat of antibiotic resistance (ABR) is growing at an alarming rate. The Centers for Diseases Control and Prevention (CDC) report more than 2.8 million antibiotic-resistant infections in the United States each year [1]. This is associated with high mortality with more than 35,000 related deaths [1]. In Europe, more than 33,000 deaths are due to antibiotic-resistant infections annually [2]. Despite limited laboratory capacity to monitor ABR; available data suggest that Africa shares the trend of increasing bacterial resistance. It has been estimated that by 2050, 10 million people will die each year from a resistant infection with more than 4 million in the African continent [3].

Any use of antibiotics, even appropriate, contributes to the development of resistance. Emergence of ABR is a natural phenomenon in response to selection pressure imposed by an antibiotic, which allows selection of strains able to resist the action of that antibiotic. However, inappropriate use of antibiotics, which is associated with increased antibiotic resistance, can adversely impact the clinical outcome of patients. Infections due to resistant bacteria are associated with severe disease, increased risk of complications and hospital admission and higher mortality rate [4, 5]. Inappropriate use of antibiotics corresponds to the use of antibiotics without clinical indications, use of an antibiotic not recommended for a condition, incorrect dose, duration and/or route of administration. The main drivers of inappropriate use of antibiotics at the health care worker (HCW)-level, include: (i) inadequate education of HCWs, (ii) prescriber’s knowledge, attitudes and practices, (iii) pharmaceutical promotion, and (iv) patient–doctor interaction [6].

The overall goal of mitigating ABR is to preserve the effectiveness of antibiotics. Health care workers have an essential role to play in antibiotic stewardship, but their role may be jeopardized if their knowledge or practices are sub-optimal. CDC estimates that 30% (47 million antibiotics courses) of all antibiotics prescribed each year in outpatient clinics and emergency departments in the US are unnecessary [7]. A study on medicines prescribed in nine US nursing homes showed that at least half of the antibiotic prescriptions contained the wrong drug, dose, or duration [8]. This figure is not better in low- and middle-income countries (LMICs) where nearly half of patients with acute viral upper respiratory tract infections and viral diarrhoea are treated unnecessarily with antibiotics [9, 10]. A study assessing knowledge of antibiotic use in Democratic Republic of Congo (DRC) showed that 39% of HCWs had low knowledge scores and less than half of respondents could accurately estimate local resistance rates for *Salmonella typhi* and *Klebsiella* spp. [11]. If patients are to use antibiotics properly, HCWs need to counsel them correctly. Appropriate use of antibiotics has been associated with lower antibiotic consumption and reduced incidence of antibiotic-resistant infections [12, 13]. In Cameroon, antibiotics make up 45–70% of prescriptions but data on their use are lacking [14, 15]. Here, we report on the knowledge, attitudes, and perceptions of medical doctors towards antibiotic use and ABR in Cameroon with the goal of identifying gaps to be addressed to optimize antibiotics use.

## Methods

### Procedure

We conducted a cross-sectional survey from May 2018 to February 2019, targeting all medical doctors practicing in major tertiary hospitals of Yaoundé, Cameroon, namely General Hospital (YGH), Gynaeco-Obstetric and Paediatrics Hospital (YGOPH), Central Hospital (YCH), and University Teaching Hospital (YUCH). The tertiary hospitals together employ approximatively 260 medical doctors including specialists, registrars, and general practitioners.

A convenience sampling was used. An email invitation generated from Research Electronic Data Capture (REDCap) tools [16] hosted at University of Cape Town-Department of Medicine, was sent to every doctor working in these hospitals. The purpose of the study was clearly explained in the aforementioned invitation email. The email was automatically resent after 10 days if there was no answer or if the questionnaire was not yet completed. This follow up period was subsequently reduced to 5 days after an initial low response rate. The invitation was resent up to five times.

The self-administered questionnaire comprised 54 questions and ten clinical cases structured in four sections:

- Socio-demographics,
- Perceptions on the importance of ABR and the awareness about causes of ABR,
- Attitudes and practices in the choice of antibiotics and potential interventions to improve antibiotic use,
- Knowledge on ABR and antibiotic use rated based on ten clinical cases on: infection prevention and control (IPC), appropriate prescription of antibiotics (community-acquired infections, extended-spectrum β-lactamase (ESBL) Gram-negative infections), duration of therapy, de-escalation, and adverse effects of antibiotics.

The questions have been made simple and unambiguous. The questionnaire was anonymous and was in English and French, the two official languages of Cameroon. The choice of the clinical cases and micro-organisms were based on the prevalence/frequency of the diseases in the selected hospitals irrespective of the wards’ specificities and are similar to the questions used in previous studies on knowledge, attitudes and practices on antibiotics use and antibiotics resistance [17].

### Ethical considerations

Electronic informed consent was obtained from each participant. All procedures were approved by both the Human Research Ethics Committee of the University of Cape Town (HREC REF: 203/2018), South Africa, and the Ethics Committee for Human Health Research in the Centre Region, Cameroon (REF: 0548/CRERSHC/2018). The study was performed in accordance with the Declaration of Helsinki.

### Statistical analysis

Descriptive statistics were used to characterize the total sample. Continuous variables were described using mean with standard deviation or median with interquartile range depending on the distribution of data. Proportions were calculated for categorical variables. Composite scoring of perceptions and attitudes was based on the five-point Likert-type response scales: strongly agree/agree/neutral/disagree/strongly disagree, strongly influence/influence/neutral/do not influence/do not influence at all, often/sometimes/rarely/never/not familiar, and very helpful/helpful/neutral/not helpful/not helpful at all [18]. Likert-type ratings (single survey questions) were summarised as proportions by collapsing the two categories on either side of the neutral category and creating an ordinal variable with three categories. The reported categories were therefore a positive response, a neutral response, or a negative response (e.g., agree/neutral/disagree). For analysis of knowledge, each correct response was scored 1 while a wrong or doubtful response 0. The mean knowledge score was then calculated. The association between the knowledge scores and composite scores of perceptions and attitudes expressed as Likert-type categories or baseline socio-demographic characteristics were compared using either one-way ANOVA test for variance or two-sample t-test respectively. Chi-square or Fisher’s Exact tests were used for assessing the differences between socio-demographic variables and perceptions/attitudes questions expressed as Likert-type categories. A p-value < 0.05 was considered as statistically significant.

## Results

### Socio-demographics

Of the 206 doctors invited to participate to the study, 98 (48%) responded and 66% (65/98) completed the questionnaire in its entirety. The median age was 31 years (interquartile range (IQR) 29–34) and 55% were female. The majority of respondents were residents (60%) (Table 1).

**Table 1:** Socio-demographic characteristics of participants (N = 98)

Characteristic	Distribution
Age in years, median (IQR)	31 (29–34)
Gender, n/N (%)	
Male	44 (45)
Female	54 (55)
Cadre category, n/N (%)	
General practitioner	11 (11)
Resident	59 (60)
Specialist	24 (24)
Unstated	4 (4)
Years as a medical doctor, median (IQR)	3 (2–6)
Years of residency, n/N (%)	
1st year resident	4/59 (7)
2nd year resident	27/59 (46)
3rd year resident	13/59 (22)
4th year resident	15/59 (25)
Years as specialist, n/N (%)	
1–3 years	14/24 (58)
4–6 years	7/24 (29)
7–9 years	3/24 (13)
Cadre speciality, n/N (%)	
Cardiology	18/81 (22)
Nephrology	9/81 (11)
Obstetrics and gynaecology	7/81 (9)
Endocrinology	6/81 (7)
Internal medicine	6/81 (7)
Surgery	6/81 (7)
Paediatrics	4/81 (5)
GIT	3/81 (4)
Pathology	2/81 (3)
Anaesthesia	1/81 (1)
Ophthalmology	1/81 (1)
ENT	1/81 (1)
Oncology	1/81 (1)
Neurology	1/81 (1)
Infectious diseases	1/81 (1)
Pulmonology	1/81 (1)
General practitioner	10/81 (12)
Unstated	3/81 (4)
Hospital, n/N (%)	
General Hospital	28 (29)
Gynaeco-Obstetric and Paediatric Hospital	6 (6)
Teaching Hospital	14 (14)
Central Hospital	40 (41)
Unstated	10 (10)

*GIT* gastro-intestinal tract; *ENT* ear nose and throat; *SD* standard deviation; *IQR* interquartile range

### Knowledge

The overall mean score on the knowledge questionnaire was 56% (± 14), with over a quarter (26%, 17/65) of participants scoring less than 50%. Participants scored well regarding the use of antibiotics for common infections—community acquired pneumonia (CAP) 71%, urinary tract infection (85%)—but scored poorly on antibiotic de-escalation (14%, 9/65) and duration of antibiotic prophylaxis (25%) (Table 2). Regarding the management of ESBL-*E. coli* infection, the choice of antibiotic was appropriate for 42% (27/65) of respondents.

**Table 2:** Summary of knowledge questions and answers

Topic	Correct answer n/N (%)
Contact precautions	42/66 (64)
Antibiotic therapy timing	64/66 (97)
Assessing severity and management of CAP	47/66 (71)
Management of acute diarrhoea	33/66 (50)
Antimicrobial selection for ESBL *E. coli*	27/65 (42)
Management of uncomplicated UTI	56/66 (85)
Interpretation of antibiogram and de-escalation	9/65 (14)
Peri-operative antibiotic prophylaxis	16/65 (25)
Antibiotics stop/review date	55/66 (83)
Management of *C. difficile*	29/66 (44)

*CAP* community-acquired pneumonia; *ESBL* extended spectrum beta-lactamases; *UTI* urinary tract infection

When evaluating factors influencing doctor’s knowledge, we found no correlation between the mean knowledge score and a previous training on antibiotic use and resistance. The knowledge score was not influenced by the doctor’s category, specialty, age, experience, or practice location (hospital, ward). Moreover, perceptions on antibiotic use and ABR were not associated with knowledge. Participants who do not mainly rely on textbooks as resources for antibiotics prescribing scored better than those who do (58% ± 13 vs 40% ± 12; p = 0.01) (Table 3). Doctors who were not influenced by patient pressure for antibiotic prescribing had a better knowledge score than those influenced by patients or those who were neutral (67% ± 12 vs 53% ± 13 or 62% ± 13; p = 0.01).

**Table 3:** Factors influencing knowledge

Characteristic	Knowledge score	*p* value
Professional category, mean (± SD)		
GP	49 (15)	0.16
Resident	56 (14)	
Specialist	61 (14)	
Hospital, mean (± SD)		
General Hospital	59 (13)	0.64
Gynaeco-Obstetric and Paediatrics Hospital	58 (13)	
Teaching Hospital	58 (13)	
Central Hospital	54 (15)	
Experience as medical officer mean (± SD)		
≤ 3 years	53 (15)	0.19
> 3 years	58 (13)	
Experience as specialist, mean (± SD)		
≤ 3 years	60 (14)	0.53
> 3 years	64 (11)	
Years of residency mean (± SD)		
≤ 2 years	54 (13)	0.38
> 2 years	58 (14)	
Previous training on dosing and duration of antibiotic therapy, mean (± SD)		
Yes	57 (14)	0.83
No	56 (14)	
Previous training on principles of infection prevention and control, mean (± SD)		
Yes	58 (17)	0.54
No	56 (14)	
Personal previous experience, mean (± SD)		
Do not influence at all/do not influence	56 (14)	0.61
Neutral	52 (15)	
Influence/strongly influence	60 (19)	
Perceptions and attitudes		
Patient reassurance, mean (± SD)		
Do not influence at all/ do not influence	67 (12)	*0.01*
Neutral	62 (13)	
Influence/strongly influence	53 (13)	
Antibiotic resistance profile in the hospital/ward, mean (± SD)		
Do not influence at all/ do not influence	56 (13)	0.07
Neutral	60 (14)	
Influence/strongly influence	40 (17)	
Risk of missing an infection, mean (± SD)		
Do not influence at all/ do not influence	55 (14)	0.27
Neutral	54 (14)	
Influence/strongly influence	61 (13)	
Patient immunocompromised or critically ill, mean (± SD)		
Do not influence at all/ do not influence	57 (14)	0.27
Neutral	57 (15)	
Influence/strongly influence	43 (15)	
Pharmaceutical firms pressure, mean (± SD)		
Do not influence at all/ do not influence	56 (12)	0.95
Neutral	57 (14)	
Influence/strongly influence	56 (15)	
Antibiotic overuse in Cameroon, mean (± SD)		
Strongly disagree/disagree	56 (14)	0.51
Neutral	45 (7)	
Agree/strongly agree	60 (-)	
Antibiotic overuse in the hospital, mean (± SD)		
Strongly disagree/disagree	56 (15)	0.66
Neutral	58 (13)	
Agree/strongly agree	54 (13)	
Antibiotic overuse in ward*		0.30
Strongly disagree/disagree	60 (13)	
Neutral	56 (15)	
Agree/strongly agree	54 (14)	
Antibiotic resistance in Cameroon		
Strongly disagree/disagree	56 (14)	0.88
Neutral	60 (20)	
Agree/strongly agree	57 (6)	
Antibiotic resistance in hospital		
Strongly disagree/disagree	57 (14)	0.38
Neutral	53 (14)	
Agree/strongly agree (11)	59 (12)	
Antibiotic resistance in ward		
Strongly disagree/disagree	57 (14)	0.95
Neutral	56 (14)	
Agree/strongly agree	56 (14)	
Resources used for antibiotic prescribing		
Infectious diseases specialist, mean (± SD)		
Not familiar/never	57 (13)	0.69
Rarely	55 (16)	
Sometimes/Often	53 (14)	
Other consultants, mean (± SD)		
Not familiar/never	55 (13)	0.73
Rarely	58 (15)	
Sometimes/Often	54 (18)	
Other colleagues, mean (± SD)		
Not familiar/never	55 (15)	0.51
Rarely	58 (12)	
Textbooks, mean (± SD)		
Not familiar/never	58 (13)	*0.01*
Rarely	40 (12)	
WHO guidelines, mean (± SD)		
Not familiar/never	57 (14)	0.73
Rarely	54 (16)	
Sometimes/Often	50 (0)	
Learned society guidelines, mean (± SD)		
Not familiar/never	57 (13)	0.21
Rarely	48 (24)	
iPad/smart phone apps, mean (± SD)		
Not familiar/never	55 (14)	0.56
Rarely	59 (15)	
Sometimes/Often	58 (13)	
Ward round		
Not helpful at all/not helpful	55 (14)	0.52
Neutral	61 (11)	
Helpful/very helpful	60 (-)	
Conferences		
Not helpful at all/not helpful	56 (14)	0.52
Neutral	60 (10)	
Pharmaceutical representatives		
Not helpful at all/not helpful	60 (12)	0.15
Neutral	53 (16)	
Helpful/very helpful	52 (12)	

*GP* general practitioner; *SD* standard derivation

### Education and source of information

When assessing common resources used by participants for antibiotics prescribing, we found that the most used were, in decreasing order of frequency, textbooks (93%, 68/73), professional/learned societies’ guidelines (93%, 68/73), WHO (World Health Organization) guidelines (81%, 59/73) and smart phones apps (60%, 44/73). Our results revealed that Infectious Diseases (ID) specialists are used as resources for antibiotic prescribing by only 58% (42/73) of doctors surveyed. Most respondents rely on other colleagues (73%, 53/73) and consultants (54%, 39/72) were the least utilised resource when prescribing antibiotics.

During the last 12 months, respondents attended a lecture or workshop or course addressing the following topics: diagnosis of infection (49%, 34/70), dose and duration of antibiotic therapy (43%, 30/70), indications for antibiotic use (34%, 24/70), spectrum of activity of commonly used antibiotics (23%, 16/70), indications for intravenous antibiotic use (21%, 15/70) and principles of Infection Prevention and Control (19%, 13/70). Nearly all participants agreed that medical journals (97%, 69/71) and conferences (93%, 66/71) are useful resources for learning on antibiotic use and resistance. Pharmaceutical representatives were considered useful resources by 44% (31/71) of respondents. It was almost unanimously felt (97%, 73/75) that development of local guidelines would be useful in improving antibiotic use and mitigating ABR. Furthermore, almost all respondents (99%, 74/75) were of the opinion that more education on antibiotic use and resistance would be helpful.

### Attitudes and perceptions on antibiotic use and antibiotic resistance

**Fig. 1:**
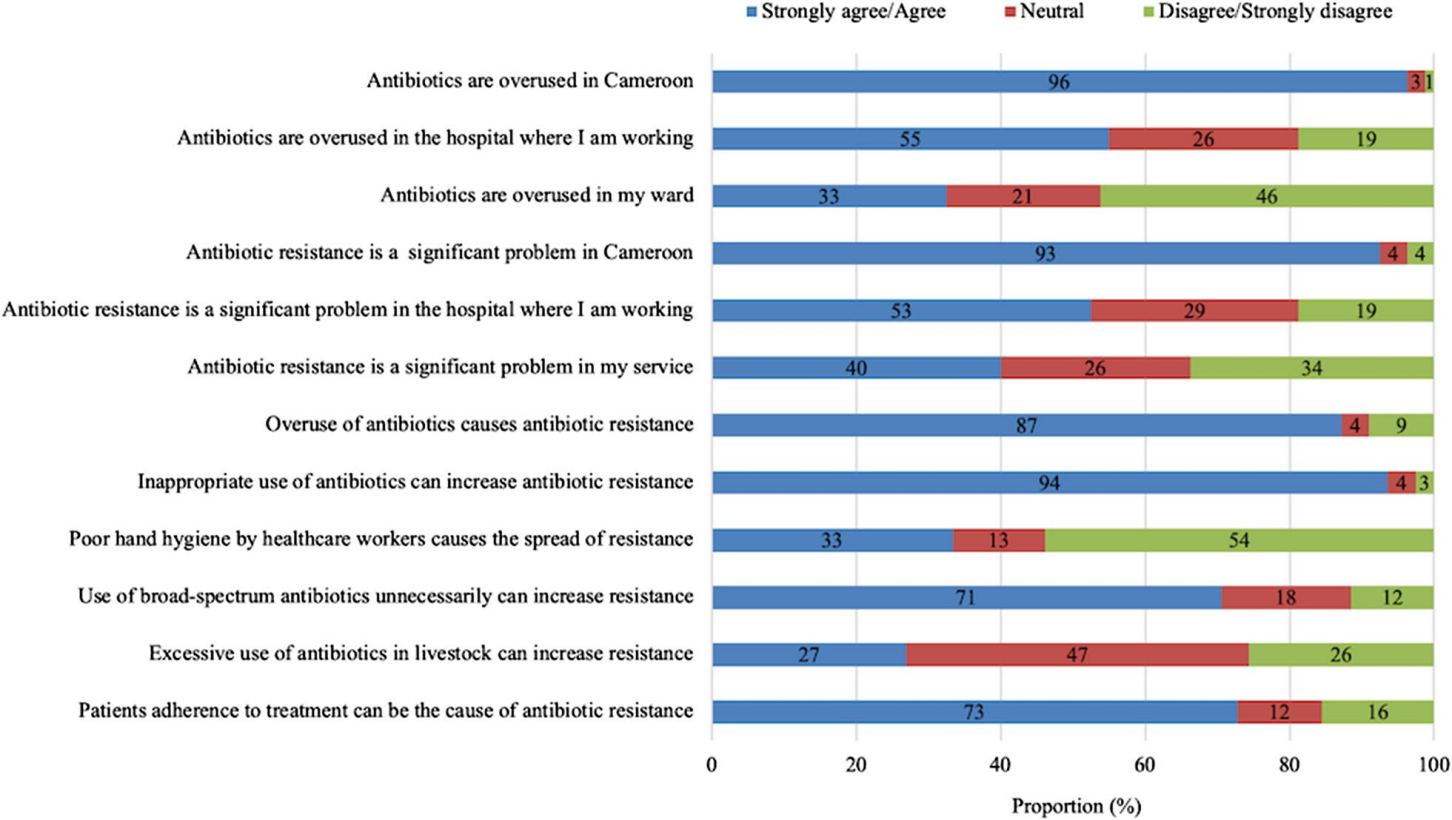
Perceptions on antibiotic use and antibiotic resistance

Most respondents, 93% (74/80) agreed that ABR is a significant problem in Cameroon, but only 40% (32/80) believed that it is a problem in their hospital wards. Interestingly, 54% (42/78) of doctors disagreed that poor hand hygiene is a cause for spread of antibiotic resistant bacteria (Fig. 1).

**Fig. 2:**
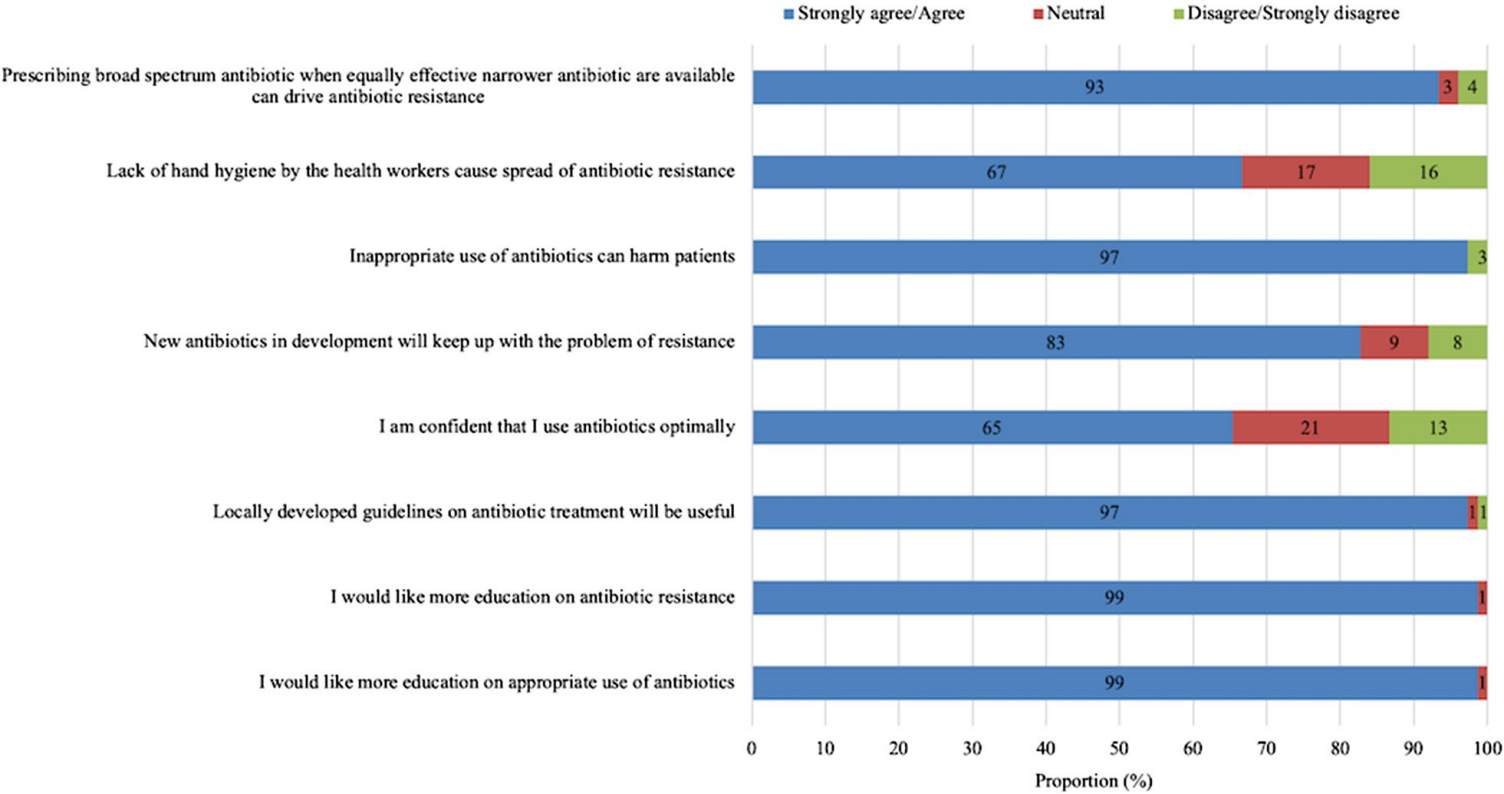
Attitudes of prescribers on antibiotic use and antibiotic resistance

Despite acknowledgement by 96% (77/80) of participants that antibiotics are overused in Cameroon, only a third believed that antibiotics are overused in their ward (Fig. 1). Two-thirds (65%, 49/75) of prescribers were confident that they use antibiotics optimally (Fig. 2). This confidence was not associated with the category of the prescriber, age, experience or previous education on antibiotic use, perceptions of antibiotic use and ABR, or level of knowledge.

Almost all respondents (97%, 73/75) agreed that inappropriate antibiotic use can harm patients (Fig. 2).

The leading factors influencing antibiotic prescribing were guidelines from learned societies and WHO (100%), quality of drug and cost effectiveness ratio (99% and 96% respectively), immunosuppression and/or critical clinical status of the patient (90%) (Table 4). Interestingly, patient expectations did not influence antibiotic prescription for 71% of doctors.

**Table 4:** Factors influencing antibiotic use (N = 73)

Parameters/Attitude	Strongly influence/influence n (%)	Neutral n (%)	Do not influence at all/do not influence n (%)
Personal previous experience	61 (84)	7 (10)	5(7)
Guidelines	73 (100)	0	0
Patient expectation	14 (19)	7 (10)	52 (71)
ABR profile in the ward	53 (73)	16 (22)	4 (6)
Risk of missing an infection	46 (63)	12 (16)	15 (21)
Patient critically ill/immunocompromised	66 (90)	3 (4)	4 (6)
Pharmaceutical company pressure	46 (63)	16 (22)	11 (15)
Cost/effectiveness ratio	70 (96)	1 (1)	2 (3)
Quality of drug	72 (99)	1 (1)	0

## Discussion

This study presents the first analysis of the knowledge, attitudes, and perceptions of physician’s use of antibiotics at tertiary level hospitals in Cameroon. Awareness on antibiotic use and drivers of resistance are a critical first step to assist prescribers in using antibiotics appropriately and optimally.

Participants in this study demonstrated a low level of knowledge and inaccurate perceptions on appropriate antibiotic use and ABR. These results are in keeping with previous studies in LMICs [11, 19]. In a country where 45–70% of drug prescriptions include at least one antibiotic, the absence of national and local guidelines on antibiotic prescribing together with insufficient HCWs training on antibiotic use and ABR could explain these findings [14, 15]. Moreover, there is a lack of ID specialists even at the higher level of health institutions. Medical doctors working in tertiary level hospitals are supposed to be the most trained on appropriate antibiotic prescribing and ABR; they are also supposed to contribute to train other HCWs at different level of healthcare institutions.

Drivers of ABR include lack of education of HCWs, beliefs, attitudes, and knowledge of HCWs on appropriate antibiotic use and ABR among others [6]. Most respondents in this study consider that issues of antibiotic overuse and ABR are more important nationwide than in their daily practice. This externalisation of responsibility, reported in several studies, can potentially maintain antibiotic misuse as physicians might be reluctant to change their habits [19, 20]. Regardless of their knowledge score, most participants feel confident that they use antibiotics optimally. This high level of confidence has been reported in similar studies with senior doctors feeling more confident than junior doctors in antibiotics prescribing [21]. We found that the level of confidence was not associated with age, experience, or specialty. High level of confidence could explain the externalisation of responsibility regarding ABR and could be a driver of antibiotic overuse. These data emphasize the need for more education with effective strategies on behavioural changes.

Encouragingly, whereas a study conducted in DRC found that the majority of respondents disagreed that inappropriate use of antibiotics can harm patients, two thirds of doctors in our cohort perceived this as a threat to patients’ safety [11]. Inappropriate use of antibiotics has been associated with selection of drug-resistant bacteria, leading to severe infections, delayed clinical response, prolonged hospitalisations, and exposure to drug side effects [22].

Patient expectation has been found to influence antibiotic prescription as shown in a study on antibiotic prescription for respiratory tract infections which revealed that physicians prescribe more antibiotics when they perceive a desire from the patient [6, 23]. In the present study, only one fifth of doctors confirmed being influenced by patients, which is far lower compared to other studies [19, 24]; nevertheless, prescribers not influenced by patients’ expectations had a better knowledge score. Previous studies have shown that prescribers who are not influenced by patient pressure for antibiotic prescribing demonstrated better antibiotic prescribing practice [20, 25].

The WHO reports that “globally, most prescribers receive most of their prescribing information from the pharmaceutical industry and in many countries, this is the only information they receive” [26]. Our data show that for more than 60% of participants, antibiotic prescribing is influenced by pharmaceutical firms with only a third who had received education or training on antibiotics spectrum of activity and indications in the last year. The absence of local guidelines and regulation on HCW-pharmaceutical firms’ relationship could explain these findings. A systematic review on physicians' interaction with pharmaceutical firms and association with their clinical practices revealed that doctors exposed to pharmaceutical companies prescribed more drugs and had a suboptimal prescribing quality [27]. In developed countries, several restrictions have been set regarding remuneration of doctors or funding by pharmaceutical firms [28, 29]. However, efforts to avoid antibiotics prescribing-related perverse incentives and misconduct are needed in LMICs.

The knowledge level of the doctors in this cohort is somewhat lower than that found in similar studies conducted in DRC and USA [11, 30]. It is important to mention that about a quarter of doctors scored less than 50%. The lowest scores obtained were on management of ESBL Gram-negative infections, peri-operative prophylaxis, antibiogram interpretation and antibiotherapy de-escalation. The lack of local guidelines as well as insufficient ID specialists and training could explain these findings; all limitations that have been highlighted by participants. Specialists scored better than residents and GPs, although it did not reach statistical significance. The score was not influenced by age, experience, category, place of work or previous training on antibiotic use and ABR. Similar results have been described in previous studies where junior doctors’ knowledge on antibiotic resistance was not different to that of senior doctors [31]. Surprisingly, participants who do not use textbooks as primary resources for antibiotics prescribing scored significantly better than those who do. This is perhaps due to more up-to-date content in other tools compared to textbooks. Nevertheless, this interesting finding would need to be confirmed in a larger sample.

Limitations to this study include the small size of our cohort, due to the relatively low response rate. Although this phenomenon is inherent to web based KAP studies, it can be partly explained in our study by limited access to internet. This could limit the generalisation of our results to the entire country. As previously shown in KAP studies, the respondents in this study may have had tendency to provide ‘socially desirable’ answers. This type of studies also provides reported instead of observed practice and the interviewees might adapt their answers to the context. To minimise the potential biases, we stressed on complete respondent’s confidentiality in our survey. We have also recommended respondents to take the questionnaire individually and not as a group. We believe that studies on larger cohorts at different levels of care, and in a wider variety of HCWs are ideally needed to confirm our findings. Confirmation of these data may help inform national policies on appropriate antibiotic use and ABR in Cameroon. Further research on the most effective tools for education of HCWs with focus on increasing knowledge on ABR should be conducted.

## Conclusion

This study has highlighted gaps in the knowledge, attitudes, and perceptions toward ABR and antibiotic use of medical doctors working at the highest level of the healthcare system in Cameroon. The low level of knowledge of prescribers contrasts with their high confidence in appropriate use of antibiotics. Additionally, their non-recognition of ABR as a problem in their ward or hospital is worrisome. Establishment of local guidelines as well as antibiotic stewardship programmes and other educational tools could be a way to improve the use of antibiotics and ABR.

## Data Availability

All data generated or analysed during this study are included in the article.

## Abbreviations

ABR: Antibiotic resistance
CAP: Community-acquired pneumonia
CDC: Centers for Disease Control
DRC: Democratic Republic of Congo
ENT: Ear nose and throat
ESBL: Extended spectrum beta-lactamase
GIT: Gastro-intestinal tract
GP: General practitioner
HCWs: Healthcare workers
ID: Infectious diseases
IPC: Infection prevention and control
IQR: Interquartile range
KAP: Knowledge attitudes and practices
LMICs: Low-and-middle income countries
SD: Standard deviation
USA: United States of America
UTI: Urinary tract infection
WHO: World Health Organization
